# Supplementary material for: The PIP Peptide of INFLORESCENCE DEFICIENT IN ABSCISSION Enhances Populus Leaf and Elaeis guineensis Fruit Abscission
Source: Plants (Basel). 2019 May 30;8(6):143. doi: 10.3390/plants8060143 (PMC6630328; doi:10.3390/plants8060143)
Supplement: Supplementary file 1 [file plants-08-00143-s001.zip › Figure S2 Phenotypes for AI calculation.pdf]

Figure S2

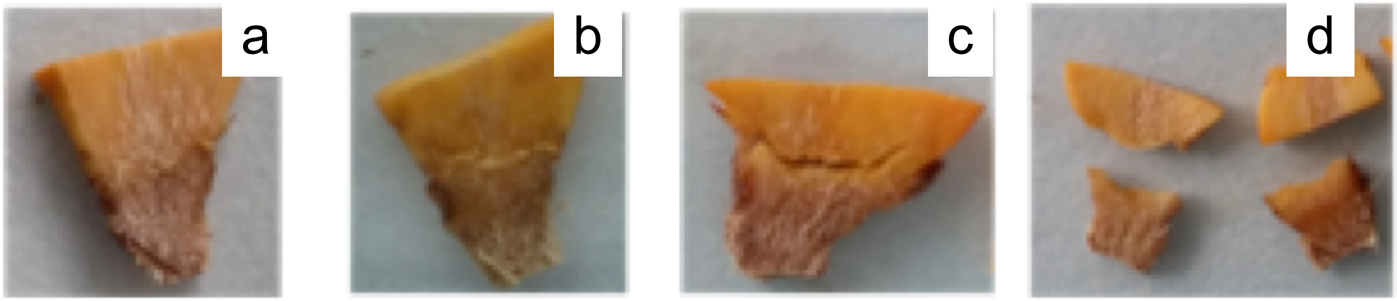


The four phenotypes defined for the test used to calculate the abscission index (AI). (a) no separation, (b) beginning to separate in primary abscission zone (AZ), (c) separation in primary AZ, (d) complete separation in both primary and adjacent AZs
