# Supplementary material for: The PIP Peptide of INFLORESCENCE DEFICIENT IN ABSCISSION Enhances Populus Leaf and Elaeis guineensis Fruit Abscission
Source: Plants (Basel). 2019 May 30;8(6):143. doi: 10.3390/plants8060143 (PMC6630328; doi:10.3390/plants8060143)
Supplement: Supplementary file 1 [file plants-08-00143-s001.zip › Figure S1 IDA Peptide methodology.pdf]

# Figure S1

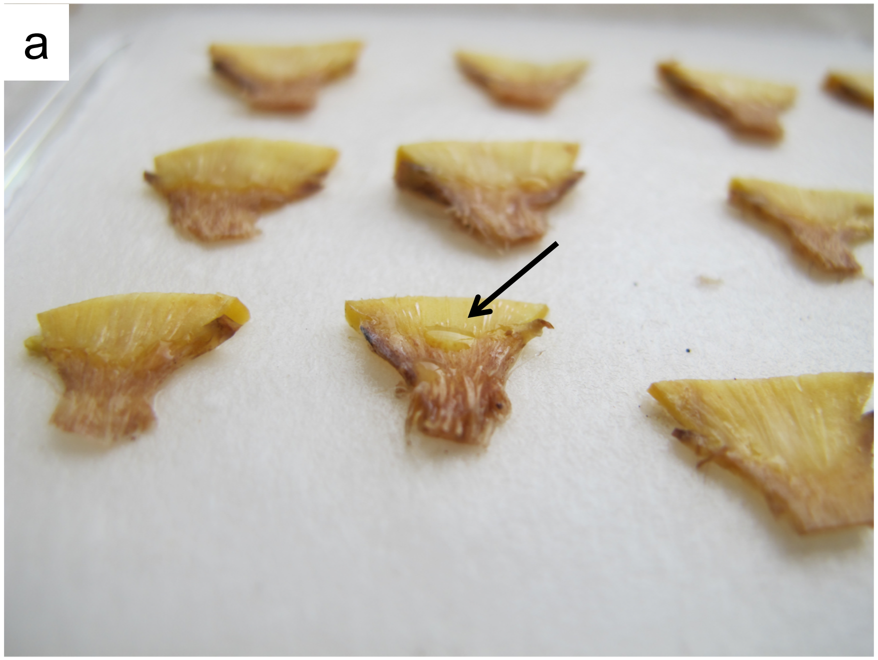


(a) Slices of fruit bases with drop of solution (arrow) with IDA peptide. Slices were incubated for approximately 24 hours at room temperature then phenotyped as described in the Materials and Methods

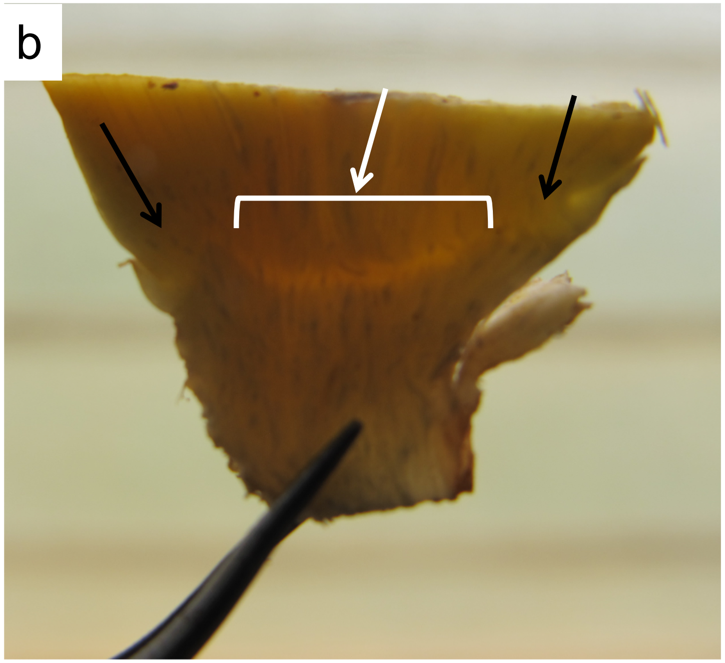


(b) Slice of the base of the oil palm fruit showing the translucent primary AZ (white arrow and bracket) and darker adjacent AZs (black arrows.
